# Transcriptomic analysis of dystrophin RNAi knockdown reveals a central role for dystrophin in muscle differentiation and contractile apparatus organization

**DOI:** 10.1186/1471-2164-11-345

**Published:** 2010-06-01

**Authors:** Mohammad M Ghahramani Seno, Capucine Trollet, Takis Athanasopoulos, Ian R Graham, Pingzhao Hu, George Dickson

**Affiliations:** 1 School of Biological Sciences, Royal Holloway - University of London, Egham, TW20 0EX, UK; 2 The Centre for Applied Genomics, Hospital for Sick Children, Toronto, M5G 1L7, Canada; 3 Wellcome Trust Sanger Institute, Wellcome Trust Genome Campus, Hinxton, Cambridge, CB10 1SA, UK

## Abstract

**Background:**

Duchenne muscular dystrophy (DMD) is a fatal muscle wasting disorder caused by mutations in the dystrophin gene. DMD has a complex and as yet incompletely defined molecular pathophysiology hindering development of effective ameliorative approaches. Transcriptomic studies so far conducted on dystrophic cells and tissues suffer from non-specific changes and background noise due to heterogeneous comparisons and secondary pathologies. A study design in which a perfectly matched control cell population is used as reference for transcriptomic studies will give a much more specific insight into the effects of dystrophin deficiency and DMD pathophysiology.

**Results:**

Using RNA interference (RNAi) to knock down dystrophin in myotubes from C57BL10 mice, we created a homogenous model to study the transcriptome of dystrophin-deficient myotubes. We noted significant differences in the global gene expression pattern between these myotubes and their matched control cultures. In particular, categorical analyses of the dysregulated genes demonstrated significant enrichment of molecules associated with the components of muscle cell contractile unit, ion channels, metabolic pathways and kinases. Additionally, some of the dysregulated genes could potentially explain conditions and endophenotypes associated with dystrophin deficiency, such as dysregulation of calcium homeostasis (*Pvalb *and *Casq1*), or cardiomyopathy (*Obscurin*, *Tcap*). In addition to be validated by qPCR, our data gains another level of validity by affirmatively reproducing several independent studies conducted previously at genes and/or protein levels *in vivo *and *in vitro*.

**Conclusion:**

Our results suggest that in striated muscles, dystrophin is involved in orchestrating proper development and organization of myofibers as contractile units, depicting a novel pathophysiology for DMD where the absence of dystrophin results in maldeveloped myofibers prone to physical stress and damage. Therefore, it becomes apparent that any gene therapy approaches for DMD should target early stages in muscle development to attain a maximum clinical benefit. With a clear and specific definition of the transcriptome of dystrophin deficiency, manipulation of identified dysregulated molecules downstream of dystrophin may lead to novel ameliorative approaches for DMD.

## Background

Duchenne Muscular Dystrophy (DMD) is a progressive and fatal muscle wasting disease, which occurs in 1 of 3500 male births worldwide [1]. DMD and related animal models such as that of the *mdx *mouse [2,3] are caused by mutations in the dystrophin gene that result in absence of the largest dystrophin isoform (Dp427) from skeletal, cardiac and smooth muscles, and the CNS [4,5]. While studies on whole animal and cell culture models of dystrophin deficiency have allowed significant progress in unraveling the molecular pathology underlying DMD, the precise pathophysiology remains poorly understood [6,7]. The cytoskeletal dystrophin protein exhibits complex interactions with many other structural and signalling molecules at the muscle sarcolemma, and its absence is associated with a diverse range of molecular and cellular disturbances [8,9]. For instance, alongside the absence of dystrophin, the stability and cellular localisation of many other proteins - the so called dystrophin associated proteins (DAPs) - are perturbed. These findings all suggest a complex primary and secondary pathophysiology associated with dystrophin deficiency.

Transcriptomic studies of diseased versus normal muscles could be very informative for understanding DMD pathophysiology. A number of groups have noted differences in the gene expression patterns between dystrophic muscle tissues of DMD patients and those of dystrophin-proficient individuals, or *mdx *compared to C57BL/6 (normal) mice [10-21]. However, studies of the direct and primary molecular sequelae of dystrophin deficiency in intact animals and tissues are complicated by secondary pathologies resulting from degenerative, regenerative, fibrotic and inflammatory changes. Cellular models of dystrophin deficiency can be complicated by non-clonal comparisons of cell lines, or by heterogeneous cellular composition (with variable myoblast, fibroblast, endothelial cell, and infiltrating blood cell content) when comparing deficient and control primary cultures. The optimum cellular model with which to evaluate the direct and primary molecular events associated with dystrophin deficiency involves a system in which expression of the protein can be manipulated experimentally in a cell autonomous manner.

RNA interference (RNAi) technology allows expression of a single gene transcript and protein product to be efficiently and specifically reduced or knocked down at the mRNA level [22]. This technique provides the opportunity to study these effects under controllable conditions, and is especially useful for multifunctional proteins such as dystrophin.

Here, we describe the specific and immediate effect of dystrophin deficiency on global gene transcription in primary muscle cell cultures in which dystrophin had efficiently been knocked down by RNAi. This approach provided the opportunity to compare the transcriptomes in dystrophin-deficient primary myotube cultures to that of their corresponding clonal cell populations, avoiding the heterogeneity routinely associated with primary cell cultures and tissue biopsies. Our results suggest that in striated myofibers, dystrophin is mainly required for proper development and organization of the contractile unit, implicating a novel pathophysiology for DMD. Genes associated with ion channels, metabolic pathways and kinases were also dysregulated in response to dystrophin deficiency. Abnormal expression of certain genes detected in our model could potentially explain conditions and endophenotypes associated with dystrophin deficiency, such as dysregulation of calcium homeostasis or cardiomyopathy.

## Results

### Transcriptome of dystrophin-deficient myotubes

**Figure 1:**
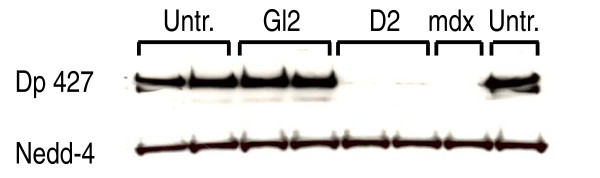
**Western blot showing strong dystrophin (Dp427) downregulation in myotubes treated with siRNA targeting dystrophin (D2)**. The samples were obtained directly from those used for gene expression arrays. Untr.: Untreated myotubes. GL2: Myotubes treated with siRNA targeting firefly luciferase. D2: Myotubes treated with an optimal siRNA targeting dystrophin (referred to as D2 in a previous study [23]). mdx: Myotubes prepared from muscles from *mdx *mice. Nedd-4: loading control.

We previously reported potent and specific knockdown of dystrophin using four different small interferring RNAs (siRNAs) [23]. Here, we used the most effective siRNA (referred to as D2) to knock down dystrophin in myotubes prepared from C57BL10 mice limb muscles. Briefly, siRNA transfections were performed at 100 nM on days two and four post-seeding and cells were collected on day six post-seeding, forty-eight hours after induction of differentiation (see **Materials & Methods **for further details). Figure 1 shows a western blot analysis of dystrophin expression in myotubes used for transcriptomic studies. This experiment showed close to zero expression of dystrophin in myotubes treated with the D2 siRNA, while this protein was well expressed both in myotubes treated with an siRNA targeting firefly GL2 luciferase (treatment control) and in untreated myotubes. No detectable change in myotube morphology was observed under phase-contrast microscopy, but immunostaining revealed an almost complete lack of dystrophin in D2 siRNA-treated myotubes (**data not shown**). In order to perform the transcriptomic analysis, we generated 7 biological replicates of primary myoblasts treated with the siRNA targeting dystrophin (D2), 7 biological replicates of primary myoblasts treated with the siRNA targeting luciferase (GL2, as treatment controls), and 4 replicates of untreated primary myoblasts (Untr.). The biological replicates were prepared and processed on 4 separate occasions, hence acting as independent replicates, too.

**Figure 2:**
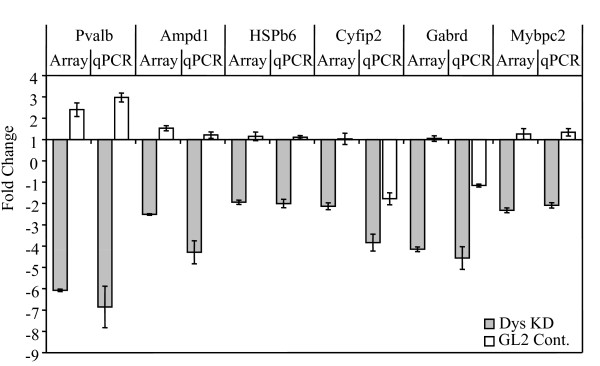
**RT-qPCR experiment supporting expression array data**. For RT-qPCR, the cDNAs made using 4 untreated biological replicates were pooled in equal amounts and used in triplicate per experiment as a reference. For each of the treatments (dystrophin knockdown (KD) or GL2 siRNA), the biological replicates from two treatment occasions (two biological replicates per each treatment occasion) were used. For each treatment occasion, the cDNAs from each biological replicate were mixed in equal amounts and used in triplicate. Relative gene expression was calculated by the ΔΔCT method. Y axis: the normalised ratio (fold change) of expression between the treated samples (Dystrophin KD or GL2 siRNA control) versus untreated samples. Error bars indicate mean +/- 1 SD.

We used Illumina gene expression arrays (Mouse whole genome-6 v1.1) to evaluate the myotubes under investigation, and analysed the data to determine the differentially expressed genes (see **Materials & Methods **for details). We made two statistical comparisons: 1) global gene expression in myotubes knocked down for dystrophin compared to the untreated myotubes (list 1); and 2) global gene expression in firefly GL2 siRNA-treated myotubes compared to the untreated myotubes (list 2). By removing from list 1 the genes in common between lists 1 and 2, we created a main list of genes changed specifically by dystrophin knockdown (Additional file 1). Then we made a final list of dysregulated genes by compiling those from the main list that changed by more than 1.5 fold (with an adjusted (Benjamini-Hochberg multiple testing corrected) *p *values less than 0.05) (Additional file 2), and those that changed in both comparisons but in different directions (Additional file 2, *italics*; see Additional file 3 for all genes common to list 1 and 2). To confirm the array data, we performed RT-qPCR on six of the dysregulated genes. As demonstrated in Figure 2, RT-qPCR confirmed gene expression changes and corroborated the array data.

Many genes of interest with respect to skeletal muscle function or the DMD phenotype can be identified amongst the dysregulated genes displayed in Additional file 2. At first glance, several genes that have been associated with various myopathies are recognised amongst the differentially regulated genes. These include kyphoscoliosis peptidase (*Ky*) [24], cofilin 2 (*Cfl2*) [25,26], calpain 3 (*Capn3*) [27], titin-cap (*Tcap*) [28,29] and phosphofructokinase (*Pfkm*) [30-32] (see Table 1 for all genes from Additional file 2 cited in the text).

**Table 1:** Gene differentially regulated by dystrophin deficiency and cited in the text

Entrez ID	Gene Symbol	Gene Name	Fold Change	Adjusted *p *value
*66211*	*Rpl3l*	*ribosomal protein l3-like*	*-6.3*	*3.80E-08*
13405	Dmd	dystrophin, muscular dystrophy	-3.7	9.54E-08
*19293*	*Pvalb*	*parvalbumin*	*-6.1*	*9.54E-08*
*78785*	*Clip4*	*CAP-GLY domain containing linker protein family, member 4*	*-3*	*9.54E-08*
*16716*	*Ky*	*kyphoscoliosis peptidase*	*-4.1*	*1.48E-07*
*59011*	*Myoz1*	*myozenin 1*	*-3.6*	*2.46E-07*
19130	Prox1	prospero-related homeobox 1	3	2.55E-05
233199	Mybpc2	myosin binding protein c, fast-type	-2.3	3.81E-05
12335	Capn3	calpain 3	-2.1	3.82E-05
*18682*	*Phkg1*	*phosphorylase kinase gamma 1*	*-3.9*	*3.82E-05*
14403	Gabrd	gamma-aminobutyric acid (gaba-a) receptor, subunit delta	-4.2	8.94E-05
243912	Hspb6	heat shock protein, alpha-crystallin-related, b6	-1.9	1.77E-04
*140781*	*Myh7*	myosin, heavy polypeptide 7, cardiac muscle	*2.7*	*2.10E-04*
21393	Tcap	titin-cap	-1.7	2.45E-04
76469	Cmya5	cardiomyopathy associated 5	-1.8	2.74E-04
380698	Obscn	obscurin, cytoskeletal calmodulin and titin-interacting RhoGEF	-2	3.19E-04
76884	Cyfip2	riken cdna 1500004i01 gene	-2.1	4.12E-04
12632	Cfl2	cofilin 2, muscle	-1.7	8.51E-04
100072	Camta1	calmodulin binding transcription activator 1	-1.6	8.96E-04
20266	Scn1b	sodium channel, voltage-gated, type i, beta	-1.7	9.09E-04
18642	Pfkm	phosphofructokinase, muscle	-1.5	1.10E-03
*11448*	*Chrne*	*cholinergic receptor, nicotinic, epsilon polypeptide*	*-3.4*	*1.16E-03*
74769	Pik3cb	phosphatidylinositol 3-kinase, catalytic, beta polypeptide	1.5	1.25E-03
12372	Casq1	calsequestrin 1	-1.6	3.27E-03
14245	Lpin1	lipin 1	-1.5	3.79E-03
109731	Maob	monoamine oxidase b	-1.5	4.95E-03
12575	Cdkn1a	cyclin-dependent kinase inhibitor 1a (p21)	-1.5	6.65E-03
*14555*	*Gpd1*	*glycerol-3-phosphate dehydrogenase 1 (soluble)*	*-1.7*	*6.65E-03*
*19309*	*Pygm*	*muscle glycogen phosphorylase*	*-1.6*	*7.55E-03*
26409	Map3k7	mitogen activated protein kinase kinase kinase 7 (Synonym Tak1)	1.5	1.22E-02
338372	Map3k9	mitogen-activated protein kinase kinase kinase 9	1.8	1.66E-02
11474	Actn3	actinin alpha 3	-1.6	1.75E-02
20661	Sort1	sortilin 1	1.6	1.82E-02

This table details genes from Additional file 2 that were cited in the text. Table sorted by ascending adjusted *p *values. *Italicized *genes are those dysregulated in dystrophin siRNA treated samples versus untreated samples, but changing in the opposite direction in the GL2 siRNA treated versus untreated samples (see result section for details).

With respect to the significance of change, dystrophin transcript is positioned to the top of the list of significantly changing genes, only next to ribosomal protein L3-like (Rpl3l). 'CAP-GLY domain-containing linker protein family, member 4' (*Clip4*) and Parvalbumin (*Pvalb*) are two other transcripts with associated *p *values similar to that of dystrophin transcript.

Ion channels [33], metabolic pathways [34-37] and kinases-related signaling pathways [38-40] are affected in dystrophin-deficient muscle cells. Several genes of each group were dysregulated in dystrophin-deficient myotubes in our experiment. For ion channels, *Scn1b *is downregulated both as reported by Pescatori *et. al. *[14] and in our experiments. Pescatori *et. al. *used muscles from children affected with DMD under the age of 2 - before any overt skeletal muscle pathology - when one would expect to see a transcriptome related to dystrophin deficiency without secondary complications. Among metabolism associated molecules, 'muscle glycogen phosphorylase' (*Pygm*), 'phosphorylase kinase gamma 1' (*Phkg1*), 'phosphofructokinase, muscle' (*Pfkm*), 'glycerol-3-phosphate dehydrogenase 1' (*Gpd1*) were reproducibly downregulated in our experiment and as reported by the aforementioned group [14]. Further, despite different species being studied, we found comparable results for 'cholinergic receptor, nicotinic, epsilon polypeptide' (*Chrne*), 'cylin-dependent kinase inhibitor 1A' (*Cdkn1a*), calpain 3 (*Capn3*), lipin1 (*Lpin1*) and 'monoamine oxidase B' (*Maob*).

Transcripts of several kinases were dysregulated in our model; amongst them, 'mitogen-activated protein kinase kinase kinase 7' (*Map3k7*), 'mitogen-activated protein kinase kinase kinase 9' (*Map3k9*) and 'phosphatidylinositol 3-kinase, catalytic, beta polypeptide' (*Pik3cb*) and 'AP2-associated kinase 1 (*Aak1*)' all upregulated. Similarly, PI3K/Akt pathway is reported to be activated in pre-necrotic (<2 weeks) diaphragm and in myotubes from *mdx *mice [38]

Heart and brain are affected in DMD, in addition to skeletal muscles. There are several genes identified in our experiment that could potentially participate in the development of heart and brain associated endophenotypes in DMD. These genes include 'Beta myosin heavy chain' (*Myh7*), Obscurin (*Obscn*), 'heat shock protein, alpha-crystalline-related B6' (*Hspb6*), titin (*Ttn*), titin-cap (*Tcap*), and myospryn (*Cmya5*) for heart associated phenotypes, and 'Cytoplasmic fragile X mental retardation (*Fmr1*) interacting protein 2' (*Cyfip2*), 'gamma-aminobutyric acid A receptor, delta' (*Gabrd*), and 'sodium channel, voltage-gated, type I, beta' (*Scn1b*) (Table 1) for brain associated phenotypes.

### Gene Ontology and Ingenuity pathway analyses

In order to have a systematic view on gene categories altered by dystrophin deficiency, we used the tool provided by the Database for Annotation, Visualisation and Integrated Discovery (DAVID) [41,42] to conduct Gene Ontology (GO) analysis on the genes listed in Additional file 2. As demonstrated in Table 2, a stringent approach to considering overrepresented GO terms (>2 fold enrichment with adjusted *p *values < 0.1) indicated enrichment for genes involved in muscle contraction, such as those contributing to sarcomere and ion channels structures. Furthermore, a less stringent but still significant (*p *< 0.05) approach revealed overrepresentation of molecules involved in metabolic pathways and kinases (Additional file 4).

**Table 2:** Gene ontology (GO) analysis of differentially expressed genes listed in the Additional file 2

Category	Term	Genes	FE	*p *val.	Adj. *p *val.
Cellular Component	GO:0044449 contractile fiber part	OBSCN, DMN, DMD, MYBPC2, TCAP, MYH13, MYH1, LDB3, ACTN3, MYBPC3, MYH7, PYGM, MYH4, TPM3,	13.83	1.74E-11	6.77E-09
Cellular Component	GO:0030017 sarcomere	OBSCN, DMN, DMD, MYBPC2, TCAP, MYH13, MYH1, LDB3, ACTN3, MYBPC3, MYH7, PYGM, MYH4, TPM3,	14.25	1.16E-11	9.06E-09
Cellular Component	GO:0030016 myofibril	OBSCN, DMN, DMD, MYBPC2, TCAP, MYH13, MYH1, LDB3, ACTN3, MYBPC3, MYH7, PYGM, MYH4, TPM3,	12.89	4.47E-11	1.16E-08
Cellular Component	GO:0043292 contractile fiber	OBSCN, DMN, DMD, MYBPC2, TCAP, MYH13, MYH1, LDB3, ACTN3, MYBPC3, MYH7, PYGM, MYH4, TPM3,	12.38	7.61E-11	1.48E-08
Cellular Component	GO:0005863 striated muscle thick filament	MYH7, OBSCN, MYH4, MYBPC2, MYH13, MYH1, MYBPC3,	36.18	1.55E-08	2.01E-06
Cellular Component	GO:0032982 myosin filament	MYH7, OBSCN, MYH4, MYBPC2, MYH13, MYH1, MYBPC3,	36.18	1.55E-08	2.01E-06
Cellular Component	GO:0005859 muscle myosin complex	MYH7, OBSCN, MYH4, MYBPC2, MYH13, MYH1, MYBPC3,	33.60	2.67E-08	2.98E-06
Cellular Component	GO:0016460 myosin II complex	MYH7, OBSCN, MYH4, MYBPC2, MYH13, MYH1, MYBPC3,	29.40	6.95E-08	6.78E-06
Molecular Function	GO:0008092 cytoskeletal protein binding	OBSCN, DMD, MYBPC2, MYH13, MYH1, ACTN3, EPB4.1L4B, MYBPC3, DCX, MTAP7, MYH7, MYH4, MYOZ1, TMOD4, PACSIN1, PSTPIP2, TPM3, SCIN, CFL2, SPNB1,	3.79	1.37E-06	3.63E-03
Cellular Component	GO:0030018 Z disc	DMN, OBSCN, PYGM, DMD, TCAP, LDB3,	13.44	7.16E-05	5.57E-03
Cellular Component	GO:0031674 I band	DMN, OBSCN, PYGM, DMD, TCAP, LDB3,	11.52	1.54E-04	1.08E-02
Cellular Component	GO:0005856 cytoskeleton	DMN, OBSCN, TTL, MYBPC2, MYH13, MYH1, ABI2, ACTN3, EPB4.1L4B, MTAP7, KRT18, TMOD4, PSTPIP2, CFL2, TPM3, DYNLRB2, GAN, DMD, LDB3, MYBPC3, DCX, MYH7, SGCG, KY, MYH4, MYOZ1, SCIN, SPNB1,	2.16	2.14E-04	1.27E-02
Cellular Component	GO:0016459 myosin complex	MYH7, OBSCN, MYH4, MYBPC2, MYH13, MYH1, MYBPC3,	8.11	2.09E-04	1.35E-02
Biological Process	GO:0003012 muscle system process	MYH7, MYH4, MYBPC2, CASQ1, KCNMA1, MYH13, MYH1, TPM3, ACTN3, MYBPC3,	7.63	5.92E-06	1.51E-02
Biological Process	GO:0006936 muscle contraction	MYH7, MYH4, MYBPC2, CASQ1, KCNMA1, MYH13, MYH1, TPM3, ACTN3, MYBPC3,	7.63	5.92E-06	1.51E-02
Molecular Function	GO:0031420 alkali metal ion binding	KCNC1, KCNA7, SCN3B, SCN1B, TTL, SLC9A7, ATP1B2, IMPA2, KCNMA1, KCNK3, SLC24A5, GMPR,	4.82	3.95E-05	5.10E-02
Molecular Function	GO:0030955 potassium ion binding	KCNC1, KCNA7, TTL, SLC9A7, ATP1B2, KCNMA1, KCNK3, SLC24A5, GMPR,	6.08	1.13E-04	7.22E-02
Molecular Function	GO:0003779 actin binding	DMD, MYBPC2, MYH13, MYH1, ACTN3, MYBPC3, MYH7, MYH4, TMOD4, PSTPIP2, TPM3, SCIN, CFL2, SPNB1,	3.77	8.93E-05	7.59E-02

DAVID tool was used for this analysis. Only terms with Benjamini-Hochberg adjusted *p *values < 0.1 and Fold Enrichment (FE) greater than 2 are displayed. Table sorted by ascending adjusted *p *values.

We used Ingenuity Pathway Analysis (IPA) (Ingenuity^® ^Systems, http://www.ingenuity.com) to further investigate the networks and functions possibly affected in dystrophic muscles. Among the genes in Additional file 2, IPA identified 229 network-eligible genes and suggested 14 main gene networks as being dysregulated by dystrophin deficiency (Additional file 5). Moreover, IPA discovered very significant enrichment (adjusted *p *value = 0.0006) of molecules involved in muscle contraction as well as significant enrichment (adjusted *p *value < 0.05) of other molecules involved in muscle (skeletal and cardiac) functions and disorders, metabolism of carbohydrates and neurological disorders amongst differentially expressed genes in our model (Table 3). IPA also indicated enrichment (adjusted *p *value < 0.05) of genes involved in actin cytoskeletal and calcium signaling canonical pathways (Table 4).

**Table 3:** Top functions and disorders predicted by IPA to be affected by dystrophin deficiency

Function or Disorder	Molecules	Adjusted *p *value
contraction of muscle	ACTN3, CASQ1, CHRNE, CKMT2, EGF, GJA5, HSPB6, KCNMA1, MYBPC1, MYBPC2, MYBPC3, MYH1, MYH7, MYLK2, TMOD4	6.33E-04
atrophy of muscle cells	ACHE, FBXO32, PPARGC1A	1.27E-02
skeletal and muscular disorder	ACHE, ACTN3, AIG1, ALKBH8, ARPP-21, BAALC, BLNK, CACNA2D1, CAMK2B, CAMTA1, CAPN3, CASQ1, CDC42BPA, CDH13, CDKN1A, CENPM, CFL2, CHRNE, CLIP4, COL23A1, CYFIP2, DCLK3, DMD, DNAJB6, GABRD, GAN, GIGYF2, GPD1, HTR2B, IL12A, IMPA2, IP6K3, KCNC1, KCNMA1, KCNN3, LDB3, LPIN1, MAOB, MLF1, MLLT3, MYBPC1, MYH7, MYH8, MYT1L, NDRG2, NOG, PADI2, PFKM, PKD1L1, PPARGC1A, PVALB, RCSD1, SCN1B, SCN3B, SERPINB1, SGCG, SORT1, SPTB, STARD10, TBC1D4, TCAP, TNRC6B, TP53BP1, TPM3, UCK2, WBSCR17, WFDC1, XK	1.46E-02
cardiovascular disorder	ABI2, ACHE, ACTN3, ARHGAP20, BLNK, CACNA2D1, CAMK2B, CAMTA1, CDC42BPA, CDH13, CDKN1A, CHRNA10, CHRNE, COL23A1, CSRNP3, CYFIP2, DMD, ENTPD3, FAM65B, GABRD, GAN, GJA5, GRHL1, HERC1, HSD11B1, HTR2B, IL12A, IMPA2, KCNA7, KCNC1, KCNK3, KCNMA1, KCNN3, KIAA1409, MALL (includes EG:7851), MAOB, MLF1, MSI2, MYBPC1, MYBPC3, MYH7, MYH8, MYLK2, NPPB, PPARGC1A, PSTPIP2, PTPRO, RCSD1, SCN1B, SGCG, SLC46A3, SLC9A2, SPTB, TBC1D4, TCAP, TNRC6B, WBSCR17, XK	2.07E-02
cardiomyopathy	GJA5, HTR2B, MYBPC3, MYH7, MYLK2, NPPB, PPARGC1A, SGCG, TCAP	2.07E-02
variant angina	CACNA2D1, RCSD1, SGCG	2.07E-02
Huntington's disease	ACTN3, AIG1, ARPP-21, CAMK2B, CASQ1, CYFIP2 (includes EG:26999), GABRD, GPD1, KCNN3, MAOB, MLF1, MYH7, MYT1L, PFKM, PPARGC1A, PVALB, SCN1B, SCN3B, SORT1, STARD10, TPM3, UCK2	3.09E-02
disease of muscle	CHRNE, FBXO32, MYH1, MYH4, PPARGC1A, SGCG, TPM3	4.23E-02
fatigue	ACHE, CHRNE, PPARGC1A, SCN1B	4.23E-02
developmental disorder of muscle	FBXO32, MYH1, MYH4, SGCG, TPM3	4.39E-02
pervasive developmental disorders	ACHE, CHRNA10, CHRNE, GABRD, HTR2B, SCN1B, SCN3B	4.39E-02
deformation of nucleus	CDKN1A, MAP7	4.39E-02
metabolism of carbohydrate	CMAH, GPD1, IMPA2, IP6K3, NISCH, PDK4, PFKM, PHKG1, PPARGC1A, PPP1R3C, PYGM, SGSH, SOCS4, ST3GAL6	4.39E-02
morphogenesis of cardiac muscle	MYBPC3, MYH7, MYLK2, TCAP	4.63E-02
muscular dystrophy	CAPN3, DMD, MYH7, SGCG, TCAP	4.69E-02
neurological disorder	ACHE, ACTN3, AIG1, ALKBH8, ARPP-21, BAALC, BLNK, CACNA2D1, CAMK2B, CAMTA1, CAPN3, CASQ1, CATSPER4, CDC42BPA, CDH13, CHRNA10, CHRNE, CLIP4, CNKSR1, COL23A1, CYFIP2, DFNA5, DMD, DNAJB6, DNAJC5, EGF, EYA3, FZD3, GABRD, GAN, GDAP1, GIGYF2, GPD1, HTR2B, IGFN1, IL12A, IMPA2, KCNC1, KCNMA1, KCNN3, KIAA1409, KPNA6, LRCH1, MAOB, MLF1, MLLT3, MSI2, MT3, MYH7, MYH8, MYT1L, NDRG2, OPN3, P2RY2, PADI2, PFKM, PKD1L1, PLA2G4E, PPARGC1A, PPP1R14C, PVALB, SCN1B, SCN3B, SERPINB1, SGCG, SORT1, SPTB, STARD10, SUSD4, TMEM108, TPM3, TTC7B, TTLL7, UCK2, WBSCR17, WWC1, XK	4.80E-02
developmental process of muscle	ACHE, CAPN3, CDKN1A, DMD, KY, MYBPC3, MYH7, MYLK2, MYOZ1, PDK4, SGCG, TCAP	4.80E-02

Genes listed in the Additional file 2 were used for this analysis. Only functions and disorders with Benjamini-Hochberg corrected *p *values < 0.05 are displayed.

**Table 4:** Top predicted (by IPA) canonical pathways to be affected in dystrophin-deficient muscle cells

Canonical Pathways	Molecules	Adj *p *value
Actin Cytoskeleton Signaling	MYH4, ABI2, CYFIP2, MYH8, CFL2, MYLK2, EGF, PIK3CB, MYH7, ACTN3, MYH1	2.45E-02
Calcium Signaling	MYH4, TPM3, MYH8, CASQ1, CHRNE, MYH7, CHRNA10, CAMK2B, MYH1	3.390E-02

Only Pathways with Benjamini-Hochberg corrected *p *values under 0.05 are displayed.

### miRNAs at the dysregulated genes loci

To evaluate possible indirect microRNA (miRNA) dysregulation by dystrophin deficiency, we investigated the possible presence of miRNA loci at the intronic regions of the genes displayed in Additional file 2. Table 5 details the miRNAs identified to happen at the dysregulated genes loci. miRbase miRNA data base (Sanger Inst., UK [43]) Release 12 was used for this analysis.

**Table 5:** Genes from Additional file 2 with miRNA loci at their intronic regions

Gene	miRNA
Arpp21	mmu-miR-128-2
Dusp19	mmu-miR-684-1
Klf9	mmu-miR-1192
Myh7	mmu-miR-208b
R3hdm1	mmu-miR-128-1

miRbase miRNA database, Release 12 was used for this analysis.

## Discussion

Despite much effort toward disentangling the molecular basis of DMD pathophysiology, it remains unclear how the absence of one molecule results in a complicated phenotype such as DMD. This is partly due to the complexity of dystrophin interactions in muscle cells. As shown by others and in the present work, the absence of dystrophin starts a cascade of derangements in several networks. This results in underdeveloped muscles, prone to the adverse effects of physical stresses that add another level of molecular complexity to the disorder. To capture the most direct effect of dystrophin deficiency, one has to get as close as possible to the early myogenesis stages when dystrophin naturally starts to be expressed. Having this in mind, we decided to use primary muscle cultures to study early myogenesis in the absence of dystrophin, by comparing the transcriptome of dystrophin-deficient cells to that of their normal counterparts shortly after induction of myogenesis. Similar approaches have been taken by others; however, we designed this experiment to avoid the cellular heterogeneity associated with the other studies. Accordingly, we used RNAi to knock down dystrophin in mouse primary myotubes, and compared transcriptomes in these cells to those in the same cell population but in which dystrophin had not been targeted. We have previously shown that RNAi is a robust technique for dystrophin knockdown [23]. Here, applying dystrophin RNAi to samples used for our transcriptomic studies resulted in close to zero dystrophin expression (Figure 1). We decided to analyze samples collected 48 hours after differentiation. At that time point, we did not observe any detectable change in myotube morphology or timing of fusion. We selected this early time point in order to focus on the early myogenesis events in the presence and absence of dystrophin expression, but avoid any unrelated secondary events such as myotube rupture and culture senescence. To account for possible unrelated variations, the experiment involved biological replicates prepared on four occasions (see **Materials & Methods **section). This design produced meaningful results, capable of giving mechanistic insights into the conditions and endophenotypes associated with DMD.

A systematic overview of genes listed in Additional file 2 by GO and IPA analyses indicated significant overrepresentation of genes associated with the physiology of contraction, kinases, or metabolic pathways. This indicates that dystrophin is not just a molecule providing structural support to myofibers, but its presence is crucial for proper development and function of striated muscle as a contractile unit.

Other earlier studies on dystrophin deficiency support our observation that ion channels [33], kinases [38-40] and metabolic pathways [34-37] are also affected in dystrophic muscles. Here, we provide a more comprehensive view of the genes dysregulated under these categories and indicate future lines of researches to address these pathways.

### Dysregulated genes involved in development of skeletal muscle phenotype

*Clip4 *and *Pvalb *had associated *p *values similar to that of dystrophin transcript. Though not much is known about *Clip4 *function, *Pvalb *is reported to be downregulated in DMD [44] and *mdx *[45] muscles. PVALB binds to Ca^2+ ^and its calcium buffering capacity is important for calcium homeostasis during muscle contraction when high level of calcium is released into the sarcoplasm. Dysregulation of calcium homeostasis, with increased intracellular [Ca^2+^]_i _is well documented in dystrophic muscles [46]. In line with this, Calsequestrin 1 (*Casq1*), the gene for CASQ1 calcium-binding protein located in sarcoplasmic reticulum (SR), was also downregulated in our model of dystrophin deficiency. Supportively, DMD muscles are reported to have deficiencies in this protein [47,48]. CASQ1 is believed to be essential for the normal development of the SR, and for calcium storage and regulation inside SR [49].

Kinases can initiate long range signaling activity by activating transcription factors that in turn will activate transcription of their target genes. One way for kinases to specify their local activity is by being targeted and anchored to the region where their activity is required [50]. A single molecule such as dystrophin could regulate the expression of a variety of myogenesis-related genes through its role in anchoring other molecules in the right locale. This property can help localise kinases and/or other signaling molecules to where their functions are required for proper myogenesis. For instance, dystrobrevin [51] and synemin [52], two dystrophin associated proteins [53,54], act to localise protein kinase A (PKA). Myospryn (*Cmya5*), a gene downregulated in dystrophin-deficient myotubes in our model, has binding sites for PKA, anchoring it at the costamere to localise PKA signaling at this complex region [55]. Supportively, it has been documented that dystrophin deficiency results in downregulation [19,40] and mislocalisation of myospryn in skeletal muscles, resulting in lower PKA activity [40]. Nearly two thirds of the genes dysregulated in our experiments were downregulated (220 of 333, Additional file 2) indicating possible under-activity of certain myogensis-related transcription factors normally activated by kinases. In fact, dysregulation of some transcription factors in *mdx *skeletal muscle has already been reported [56].

Our results also suggest that myofiber type fate determination may be affected by dystrophin deficiency. Upregulation of 'prospero-related homeobox 1' (*Prox1*) is a key step in formation of slow muscle fibres [57]. Our samples in which dystrophin had been downregulated showed three-fold increase in *Prox1*. Additionally, 'Fast type myosin binding protein c' (*Mybpc2*) and 'alpha actinin3' (*Actn3*) were downregulated in dystrophin deficient myotubes in our experiment. *Actn3 *levels increase in accord with increases in skeletal muscle fast fibers [58]; and specific expression of *Mybpc2 *in fast fibre muscles has also been documented [59].

### Dysregulated genes involved in development of heart phenotype

Heart is another organ which is involved by the pathogenesis of dystrophin deficiency. There are several molecules identified by our model that can potentially explain the mechanism(s) by which heart is adversely affected in DMD. For instance, obscurin, which was downregulated in our experiment, is required for proper assembly of M-band and A-band and myofibrillar clusters and for the regular alignment of the SR network around the contractile apparatus [60,61]. *Hspb6*, which associates with actin, is a cardioprotector under stressful conditions [62] and its mRNA was downregulated in association with dystrophin deficiency in this experiment.

Z-disc and costamere and their associated molecules function as mechanosensors in striated muscles, and their perturbation can result in architectural distortion of the affected muscle cells [63]. Hence, mutations in some genes involved in this area, such as titin and titin-cap, can result in cardiomyopathy [64,65]. Titin-cap was downregulated in dystrophin-deficient myotubes in our experiment, as was myozenin 1, another Z-disc associated protein.

Expression of the myospryn gene was downregulated by dystrophin deficiency in our model. It is also downregulated in both DMD [19] and *mdx *[40]; and single nucleotide polymorphisms at the myospryn locus are associated with ventricular hypertrophy [66]. This gene is expressed exclusively in heart and skeletal muscle during embryogenesis and in adult striated muscle [67]. Interestingly, as it was mentioned earlier myospryn protein is a docking molecule for protein kinase A at the costamere, and involved in localising and specifying signal transduction at this region [40].

## Conclusion

Our work depicts a specific transcriptome associated with dystrophin deficiency, with minimal noise from non-specific complications such as non-clonal cells or biopsy-associated issues such as necrosis/degeneration/regeneration. While our results are affirmatively reproducing several previous *in vitro *and *in vivo *experiments, indicating the soundness of the data, they additionally suggest that dystrophin may be crucial for proper development of striated muscles as an organized contractile unit, implicating a novel pathophysiology for DMD. Dysregulation of several molecules converging at functions associated with the contractile unit of the myofibers is indicative of an aberrant myogenesis process resulting in maldeveloped myofibers which may be susceptible to physical or cytotoxic stress. Future experiments should include time course studies to identify longer-term sequelae of dystrophin deficiency. These data complement our previous *in vivo *work with dystrophin shRNAs expressed from AAV vectors [23], which suggested that the presence of dystrophin is not as essential after striated muscles have fully developed. Accordingly, transient gene therapy or other approaches to counter dystrophin-deficiency at early stages of skeletal muscle maturation might confer long lasting therapeutic benefit. In addition, the identification of dysregulated genes linked to dystrophin deficiency will provide insights and experimental approaches to define novel therapeutic targets for the potential treatment of DMD.

## Methods

### Cell culture & siRNA transfection

Primary muscle cultures were prepared from limb muscles of 3 to 5 day old mice, as described previously [68]. Seven biological replicates of primary myoblasts treated with the siRNA targeting dystrophin, or the siRNA targeting GL2 luciferase (as treatment controls), and 4 replicates of untreated primary myoblasts were prepared on four occasions. In order to have a uniform cell population between replicates at each instance, the muscle cell homogenates from different mice were mixed well and the resulting cell mixture was used to prepare the cultures. The cells were seeded in 150 cm^2 ^tissue culture plates and one animal per plate was used. After approximately 48 hours, the cultures were about 60% confluent and were subjected to the first transfection with siRNAs. The siRNA targeting dystrophin had been designed using an algorithm developed by Cenix BioScience GmbH (Dresden, Germany) and manufactured by Ambion (Austin, USA) (see [23] for more details). The control siRNA targeting firefly GL2 luciferase was ordered from Dharmacon (Lafayette, USA). siRNAs were introduced into the primary muscle cultures at a final concentration of 100 nM using Oligofectamine transfection reagent (Invitrogen, Paisley, UK), following the manufacturer's instructions. Briefly, at the time of transfection, media were replaced with 13.5 ml per plate of complete DMEM supplemented with 10% serum. To transfect the cells in each plate 45 μl oligofectamine was gently diluted in 105 μl OptiMEM (Invitrogen) and incubated at room temperature for 10 minutes. The oligofectamine preparation was then added to 75 μl (20 μM) oligonucleotide already diluted in 1275 μl of OpiMEM, mixed gently and incubated for 20 minutes at room temperature before adding to the plates. 7 hours after transfection 15 ml of complete DMEM supplemented with 20% serum was added to each plate. 96 hours after seeding, when cultures were fully confluent, media were changed to serum-free Dulbecco's Minimal Essential Medium (DMEM) to induce differentiation of myoblasts, and a second transfection was performed at this time in serum free media as explained above. Cells were harvested for protein and total RNA assays 48 hours after the second transfection.

### RNA isolation

Total RNA was extracted using PARIS kit (Ambion) according to the manufacturer recommended protocol. The quality of RNAs was checked using Agilent Bioanalyser (Santa Clara, USA). RNAs were stored at -80°C until used.

### Antibodies

We used the following antibodies in this experiment: mouse monoclonal antibody NCL-DYS2 detecting the C-terminus of dystrophin (Clone Dy8/6C5; Novocastra Laboratories, UK), Nedd-4 mouse monoclonal antibody detecting Nedd-4 (Transduction Laboratories, USA) and Goat anti-mouse HRP-secondary antibody (Jackson ImmunoResearch Laboratories, USA).

### Western Blotting

Protein extracts were obtained by using a small portion of the same cell samples lysed for RNA isolation using a PARIS kit (Ambion) as instructed by the manual. The cell lysates were added with equal volumes of 2× lysis buffer. 1× lysis buffer contained 75 mM Tris-HCl (Sigma-Aldrich, UK) pH 6.8; 10% SDS (Sigma-Aldrich); 20% Glycerol (Sigma-Aldrich); 200 mg/ml EDTA (Sigma-Aldrich) and 20 μg/ml phenylmethylsulfonyl fluoride (Sigma-Aldrich). Cell lystes were then passed through a 25 gauge hypodermic needle several times to shear DNA and hence reduce viscosity. DTT (Dithiothreitol, Sigma-Aldrich) and bromophenol blue were excluded from the buffer in this stage as they interfered with protein assay. To determine the total protein amount in samples, the BCA protein assay kit and protocol (Perbio Science, UK) was used. Twenty micrograms of each of the samples was run on Tris-Acetate 3-8% NuPAGE gels (Invitrogen) using Tris-acetate SDS running buffer (Invitrogen) for dystrophin detection.

### RNA amplification and Illumina expression array chip hybridisation

The RNA amplification, chip hybridization and data extraction was done as a service by The Centre for Applied Genomics, Toronto, Canada. Illumina MouseWG-6_V1_1 gene expression arrays (San Diego, USA) were used for global gene expression studies. 250 ng total RNA was used to make amplified cRNAs using Illumina TotalPrep RNA amplification kit (Ambion). 1.5 μg cRNA was then hybridized to each microarray chip following manufacturer instructions. The signals were detected and gathered using BeadArray Reader (Illumina). The data were then uploaded to Bead Studio software (Illumina) and after qualitative evaluation, the text file data were extracted and statistically analyzed as explained below. Gene expression data have been deposited in Gene Expression Omnibus (GEO) http://www.ncbi.nlm.nih.gov/geo/index.cgi with the assigned GEO accession number GSE20548.

### Statistical analysis

Background correction was made as described by Irizarry et al. [69]. The background corrected data were then transformed into log2 scales, and quantile normalisation was performed between arrays as previously described [70]. The three-step normalisation procedure was carried out using the Lumi software package for the R programming environment [71]. To assess differential gene expression, LIMMA (linear models for microarray data) method [72] was used. *p *values were adjusted for multiple testing using the Benjamini and Hochberg method.

### RT-qPCR and primers

RNA samples were first treated with DNase I (Qiagen, Germany) as instructed by the manufacturer to remove possible DNA contaminations. 1 μg total RNA was then reverse transcribed using the Supercript III reverse transcription kit (Invitrogen) following manufacturer instructions. 1-2 ul of 1:5 diluted cDNA were used for quantitative PCR reaction using Brilliant II fast SYBR Green qPCR master mix (Agilent Technologies) in a total of 15 μL reaction volume. PCR reaction was carried out as follows: 10 minutes at 95°C followed by 40 cycles at 95°C for 20 s and 60°C for 60 s. Specificity of the PCR product was checked by melting-curve analysis using the following program: 55°C increasing 0.5°C in 60 steps, each step was 10 s temperature. Expression levels were calculated according to the ΔΔCt method normalised to the Elval1 mRNA expression. The *Elval1 *gene was used as an internal normaliser, as it showed the least variation (based on our microarray data) among all different replicates and conditions.

RT-qPCR primers were designed at exon-exon boundaries using Primer 3 software [73], unless mentioned otherwise (Additional file 6). The primers were initially tested by running an end-point PCR for each using SybrGreen master mix. The products were run on agarose gel for the product size as well as being used to perform melting curve analysis.

### Animal husbandry

Animals were bred in-house and food and water provided ad libitum. They were maintained, and experimentation conducted under statutory Home Office recommendation, regulatory, ethical and licensing procedures and under the Animals (Scientific Procedures) Act 1986 (PPL 70/6160).

## Abbreviations

DMD: Duchenne muscular dystrophy; RNAi: RNA interference; siRNA: small interfering RNA; IPA: Ingenuity Pathway Analysis; DAVID: Database for Annotation, Visualization and Integrated Discovery; PKA: protein kinase A.

## Authors' contributions

GD and MMGS designed the experiment. MMGS made primary cell cultures, performed RNAi, prepared the RNA, conducted qRT-PCR and performed GO and IPA analyses. CT helped with RNA and RT-qPCR data preparations. IRG helped with the cell culture and western blot experiment. TA helped with RNAi. PH performed statistical analysis of microarray data. GD, MMGS, CT, TA and PH wrote the paper. All authors read and approved the final manuscript.

## Supplementary Material

Additional file 1 **A table listing genes initially prepared from genes changing by dystrophin deficiency**. Click here for file

Additional file 2 **A table listing all genes changing by dystrophin deficiency that was used for further GO and IPA analyses**. Click here for file

Additional file 3 **A table listing changing genes common to "dystrophin siRNA treated versus untreated samples" and "GL2 siRNA treated versus untreated samples" comparisons**. Click here for file

Additional file 4 **A table listing enriched GO terms amongst genes changing by dystrophin deficiency**. Click here for file

Additional file 5 **A table listing predicted networks by IPA to be dysregulated by dystrophin deficiency**. Click here for file

Additional file 6 **A table listing primers used for RT-qPCR**. Click here for file
