# Supplementary material for: Transcriptomic analysis of dystrophin RNAi knockdown reveals a central role for dystrophin in muscle differentiation and contractile apparatus organization
Source: BMC Genomics. 2010 Jun 1;11:345. doi: 10.1186/1471-2164-11-345 (PMC2890566; doi:10.1186/1471-2164-11-345)
Supplement: Additional file 6 — A table listing primers used for RT-qPCR. [file 1471-2164-11-345-S6.DOC]

**Additional data file 6.** Primers used for RT-qPCR.

| **Gene Name** | **Forward Sequence (5’>3’)** | **Reverse Sequence (5’>3’)** | **Comment** |
| --- | --- | --- | --- |
| *Elval1* | AGACCACAGGTTTGTCCAG | ATAGGGGAGAACCTGAATCT | Exon boundaries |
| *Ampd1* | GCCTGAGAAAGGAACGAG | GCACAGGACTCTTCTTTAAATTC | Exon boundaries |
| *Cyfip2* | GTACGGCTCTCCAGGAAT | CTCCCCCTCTTTGATGTAG | Exon boundaries |
| *Gabrd* | TGGACCTAGAGAGCTATGG | ACAGTGGTGATGCCTAGAGA | Exon boundaries |
| *Hspb6* | GCCCAGGTGTCCACG | CGTGTTCATCCGGGCG | Manual design |
| *Mybpc2* | TTCTCAAGACAGGAATCACC | ACACCACCTTTGGCTTAG | Exon boundaries |
| *Pvalb* | ACAAAGACAAAAGTGGCTTC | GAATTCTTCAACCCCAATC | Exonic |
